# Agile Authorship: Exploring Collaborative Dementia Memoirs through Fanfiction Practices

**DOI:** 10.3828/jlcds.2025.45

**Published:** 2025-12-24

**Authors:** Nicola Simonetti, Marialaura Grandolfo

**Affiliations:** Durham University/Independent

## Abstract

This article examines how collaborative dementia memoirs can challenge personalised approaches to authorship in impactful ways. Collaborative life writing has traditionally grappled with the complex power dynamics inherent in multi-contributor narratives. Literary disability scholars, in particular, have raised ethical concerns about the possible exploitation and erosion of agency among disabled subjects. As an alternative, we advocate for a paradigm shift aligned with dementia self-advocates’ emphasis on interdependence, drawing on potentially productive concepts of shared authorship found in fanfiction. In fan communities, all fanfiction is perceived as oppositional to the authority of the single author, framing stories as dynamic rather than fixed to one voice. We apply this perspective to *Slow Puncture* (2020) by Peter Berry and Deb Bunt and *Somebody I Used to Know* (2018) by Wendy Mitchell with Anna Wharton, where collaboration and interdependence are central to narrating the dementia experience and negotiating both authors’ understandings of the condition, as well as facilitating self- and mutual exploration through creative production. We also propose the concept of “agile authorship” as a means to approach questions of authorship in collaborative memoirs by contributors with and without dementia.

## Introduction

After Philippe Lejeune published *Le Pacte autobiographique* in 1975, the explosion of the neat correspondence – at least of name – between author, narrator and subject of the solo autobiography has been the focus of much life writing scholarship. Lejeune’s threefold identity model has been assessed and revisited – including, among others, by Lejeune himself (“The Autobiography of Those Who Do Not Write”) – with regard to increasing practices of collaborative life writing. Attention to varying degrees of creative intervention and how collaboration is marked within the textual structure has given rise to a complex taxonomy of subgenres. Combining features of the autobiographical and biographical genre are as-told-to autobiographies, conventionally involving one person telling their life story to another, who writes in on their behalf. Near these can be found those nominally single-author texts, collaborative to some degree, which Paul John Eakin calls relational lives and G. Thomas Couser calls auto/biographies (or, in the context of illness and disability, auto/somatographies) (*Signifying Bodies*, 2). In between autobiography and biography, we would find dual autobiographies, where each partner contributes a separate narrative.

The potential of these partnerships to uncover life stories that might otherwise remain buried for various health-related reasons has not gone unnoticed. Reflecting on the bitter controversy surrounding facilitated communication for some autistic individuals, for example, Sidonie Smith describes interventionists’ perception of autism as “a silence in need of a story” (233), which can finally emerge through collaboration with trained assistants. However, more often, conversations about collaborative life writing are driven by the dangers and tensions relative to such collaborations. In *Vulnerable Subjects* (2004), Couser examines the ethical complexities surrounding this genre, including cases where there is a clear power imbalance between disabled subject and nondisabled writer. Couser carefully considers the potential for abuse in collaborative life stories, which partly stems from an issue obscured by the term itself: “that is, the process, though cooperative, does not usually involve collaborative *writing* (which can itself be problematic) […] There are, of course, different kinds and degrees of collaboration, but in most cases, one member supplies the ‘life’ while the other provides the ‘writing’” (*Vulnerable Subjects*, 36). Such a division of roles points to an issue close to the heart of collaborative life writing: “Whose property is a collaboratively produced life story?” (Couser, *Vulnerable Subjects*, 47). For Sandra Lindemann, collaborative life writing can escalate in a contest for authority between parties, battling to determine *who is the author?* and *whose story is this?*. In her words, “writers walk a tightrope between betrayal and complicity” (386).

We agree with Couser that ill or disabled people may find themselves at a disadvantage with respect to their collaborators (*Vulnerable Subjects*, 37). Couser’s discussion makes it clear that there is reason for readers and critics to pay attention to the power imbalance that can be present in collaborative life stories of illness and disability. Importantly, we acknowledge the potential ethical issues of collaborative life writing. Nevertheless, we agree with Monica Orlando about the importance of noticing “not only the pitfalls but also the potential” of this approach (218), for the way it fashions nuanced entanglements between disabled and nondisabled lives. To this end, we propose adding fanfiction studies to the array of critical discourses on collaborative life writing. In particular, we highlight the benefits of using a framework that resists thinking of authorship as individual and avoids interpreting collaboration as a hierarchised relation dominated by the nondisabled writer. Our intervention allows for a celebration of two texts such as *Slow Puncture* (2020) – a collaborative memoir of Peter Berry’s adult life with early-onset Alzheimer’s and Deb Bunt’s encounter with Peter – and *Somebody I Used to Know* (2018) – Wendy Mitchell’s memoir, co-authored with Anna Wharton, about living with the same diagnosis. Both memoirs emerge as naturally dialogical narratives, where questions of authorship and authority are complicated – but not limited – by the dynamic interplay between contributors.

After providing an overview of the possible contribution of fanfiction studies, we explore how collaborative life writing in *Slow Puncture* serves as a safe space for its contributors to negotiate their understandings of dementia, and how the progressive blending of alternating narrative strands reinforces the memoir’s integrally collaborative nature. Second, we discuss *Somebody I Used to Know* to challenge the notion that collaborative autobiography is inherently ventriloquistic (Couser, *Vulnerable Subjects*, 48) and presupposes a quite clear division of roles (Lejeune, “The Autobiography of Those Who Do Not Write”, 188-89); instead, we view the memoir’s single narrative voice as a profound expression of partnership. Both texts support a case for the potential of a new approach to authorship in collaborative dementia memoirs, which we call “agile authorship”.^1^ This concept is inspired by practices in action in fandom spaces and aligns with dementia self-advocates’ emphasis on interdependence and solidarity (Keyes et al.), nodding to disability studies’ idea that individual realisation “is not autonomy and independence but dependency and interdependence” (Davis 275).

### Authorship and/in Fanfiction

As the Introduction suggested, partnership in life writing is never easily defined. In his discussion of what he calls the autobiographies of those who do not write, referring to individuals who do not take the form of the personal life story to transmit memories, Lejeune revisits the autobiographical pact he had theorised five years earlier. He does so to consider the tensions underlying the question *who is the author?* in collaborative autobiographies, which, in turn, are problematically shaped by humanist ideas of authorship. As Lejeune claims: “This atmosphere of distrust has developed since the beginning of the nineteenth century, that is, since the role of the author has become highly personalized (since we have become used to consuming the person)” (“The Autobiography of Those Who Do Not Write,” 194). Autobiographical writing, he explains, is problematically dependent on the reader’s idea that “the model must take over the writing: the status as author is part of this *value* that the reader admires. The illustrious, or exemplary, person must be a full and complete subject” (“The Autobiography of Those Who Do Not Write,” 195). Any collaborative efforts must, therefore, be hidden.

Over the last fifty years, this ableist perception – which Lejeune problematised but often persists in the publishing industry today – has been widely challenged. Striving to adapt the autobiographical genre to describe the experience of those who either cannot write, or, more broadly, have difficulties making their life known through conventional life writing strategies, literary disability studies has uncovered and thoroughly discussed – in a reversal of trend – the partnership at the core of many life stories about disability. Terms such as as-told-to autobiographies and dual autobiographies are just two of the many expressions wanting to reconcile, oxymoronically but openly, ideas of identity between author, narrator and subject and collaborative principles of composition. It is undeniable, however, that terminological and epistemological tensions remain at the foreground of these terms. As Lindemann observes, for example, as-told-to life writing has been traditionally read as autobiography, but it can and should also be recognised as a form of biography (386). Central to these subgenres, we hold, is the idea that partners of a collaborative work of life writing retain precursive separate identities in the act of composition itself. So, the questions *whose story is this?* and *who is the author?* aim to weigh each partner’s contribution and determine which contributor should be credited for the narrative voice. “A life has only one author,” declared publisher François Maspero (14, our translation). But understanding knowledge production as cleanly emergent from an individual thinking self is a normative construct (Tembo and Bateson 532).

What we suggest in this article is a paradigm shift. While recognising the value of claiming one’s story as one’s own – poststructuralist ethnography has made this one of its core tenets – our focus is away from the practice of personalising authorship, and onto the potential of shared authorship as a democratic space of dis/ability realisation. We therefore turn to fanfiction as an explorative stance from which to look back at questions of partnership in life writing. Unlike most published literature, fanfiction operates outside traditional literary conventions and marketplace regulations. Whereas life writing partnerships, as Couser suggests in *Vulnerable Subjects*, typically depend on “the conditions and division of labor and the distribution of the proceeds” (43), fanfiction has historically been non-commercial. The product of life writing is often regarded as an individual’s possession that may yield profit and prestige. Instead, fanfiction narratives are treated as shared common goods, including those with autobiographical elements known as self-inserts, that is, stories in which original characters function as avatars of the fanfiction participants (Lehtonen 107). Camille Bacon-Smith compares fans’ creative productions to practices like quilting, gothic romances authored by women in the 1850s and commercial television: the final work is not the manifestation of individual genius or “an aesthetically unique piece of art” (57), but rather a collaborative assemblage of “infinitely combinable but specifically not unique elements” (57).

As a result, the personalised model of authorship associated with most published life writing is unfamiliar to fanfiction practices. By contrast, there is a sense that all fanfiction is inherently oppositional to the single authority of the (predominantly White male) author figure (Fathallah 127). Within what Henry Jenkins has called “participatory culture” (3), the author is – in a Barthesian sense – dead. Questions such as those raised by Lindemann are only pertinent if upholding Maspero’s belief that one life can only have one author, with the resulting life story being as original as the individual who produces it (Woodmansee 280). The originality expected in this context is not as valued in fanfiction, where repetition, appropriation and derivation are accepted and welcomed practices, not only between source text and fanfictions but also among fanfictions themselves. So, for example, contributors of self-insert fanfictions willingly give their personal experiences and perspectives to the fanfiction community to enjoy, share and rework. The *Tumblr* blog *AO3 Comment of the Day* explains this by distinguishing between plagiarism and imitation. According to the blog, plagiarism in fanfiction contexts occurs only “when someone copies the exact words of another author and then claims that they wrote those words themselves” (n.p.). Conversely, the blog argues that “[i]mitation is the sincerest form of flattery” (n.p.). This perspective underscores fanfiction’s reliance on contributors’ mutual amicability.

Collaboration in fanfiction is not indicative of vertical social relations, as Couser warns about (*Vulnerable Subjects*, 37), and differences in culture, gender, class, age or health conditions – if present – are hidden by a notable absence of proper names, with pseudonyms used in their place. What is more, the pseudonymous signature that appears on a fanfiction’s webpage – akin to the author’s signature on a book cover, which Lejeune described as irrefutable evidence of a real person in the world-beyond-the-text (“The Autobiographical Pact,” 11) – can never be reliably linked to any real-life individual. Pseudonyms provide no guarantee of concealing an individual person. For all the reader knows, they could represent the coming together of multiple contributors (as one joint co-author) or, as in the case of as-told-to life writing, an individual writing under the guidance of another.

It is instructive, in this sense, to expand the notion of writing which, as implied by its prominence in the genre’s name, has become increasingly linked to concepts of authorship and ownership in life writing. Unlike Couser’s critique of the unacknowledged mediation involved in many as-told-to autobiographies, which he compares to legal forgery (*Vulnerable Subjects*, 50), mediation is not seen as plagiarism on the part of the fanfiction writer, nor is there the belief that one’s story is only one’s own. Writing, for fanfiction contributors, is a tool of preservation rather than creation. While fanfiction may be penned by a single writer, within fandom the act of writing does not grant greater authority over the text, nor do writers necessarily believe it does. Surprisingly, Lejeune supports a similar view. Speaking about collaboration in autobiographies, he explains: “The author of a text is most often the one who wrote it, but the fact of writing is not sufficient to be declared an author. One is not an author in the absolute” (“The Autobiography of Those Who Do Not Write,” 192). So, the stories that fanfiction contributors put into writing are those that circulate in their communities, often in more fleeting forms such as discussions in group chats. Much like contributors of collaborative dementia memoirs who live without dementia, fanfiction participants frequently write narratives that do not originate from them – or at least only partially – and that others may be unable or unwilling to write. The use of pseudonyms highlights the constructed quality of the author figure. In *Fanfiction and the Author* (2017), Judith May Fathallah examines the shared showrunner role in the BBC series *Sherlock*, observing that “[w]hat matters are their [the showrunners’] positions and authorial statements, through which they present a united front and attitude to the show” (50).

This atmosphere of trust, which James Paul Gee calls an “affinity space” (67), facilitates self- and mutual exploration through creative production. Fanfictions have established themselves as spaces where individuals belonging to marginalised groups can create and exchange narratives free of judgment and discrimination. For example, Sanna Lehtonen observes how fanfiction can offer possibilities “for writers’ gendered identity work by providing a safe venue where young women writers reflect upon their life events and identities by partly fictionalizing their real-life experiences” (107). Similarly, Angela Thomas describes the collaborative journey of two adolescent girls who co-authored fanfiction, emphasising how each contributor gained distinct benefits from the partnership, which they perceived as a fundamentally joyous and enriching experience. In these cases, identities are given to be shared, and (re)constituted through relational interactions.

### *Slow Puncture*: The Transformative Power of Collaboration

As in fanfiction, the focus of our article is not on denying individuality but celebrating interdependence and exploring how this concept is reflected in attitudes towards authorship in collaborative dementia memoirs. With regard to dementia in particular, the approach we highlight aligns with dementia self-advocates’ insistence on a citizenship perspective. This perspective does not downplay the difficulties associated with dementia; rather, it stresses them to caution against the cultural overemphasis on individualism (Keyes et al.; Örulv, “Self-help, Mutual Support and Advocacy”). In believing this, many people with dementia have reaffirmed “a social perspective of being interrelated with others and with society, as agents depending on other agents, in line with a social model of disability” (Örulv, “Neurodiversity and Dementia,” 251). Collaborative dementia memoirs, as literary refractions of social relations between disabled and nondisabled people, provide fertile ground for exploring how the identities and authorial voices of people with and without dementia shape and are shaped by the text.

Advocacy for interdependence is central to *Slow Puncture*. Written by Deb Bunt, as she acknowledges in the Prologue – “We agree that I will write his book for him [Peter Berry], using his words but breathing life into them” (x) – the memoir is divided into chapters narrated alternatively from the perspectives of Peter and Deb. Rightfully, one might suggest that the text is not evenly balanced; Deb is the writer of both her chapters and Peter’s. However, we agree with Orlando that the writing process or the text “should not be seen as *less* authentically collaborative because of obstacles due to one’s party disability” (220). Instead, collaboration and collaborative composition are at the heart of *Slow Puncture*, where the two characters’ narratives intertwine and overlap, inviting a rethinking of stereotypes about people with dementia and affirming both partners as equally instrumental in the memoir’s creation. Reflecting on Peter’s story, Deb moves towards a new understanding of dementia, focusing on the potential, rather than solely on the limits, of people with this condition. Meanwhile, Deb’s writing empowers Peter, for whom collaborative writing creates a new purpose in life (27). At the core of this work is a non-hierarchical relation between the two partners, comparable to that among fans, and the idea that collaborative writing is not only a means of restoration but also a powerful tool for co-creation.

In a number of ways, Peter’s narrative before meeting Deb describes his state of being as broken and an obstacle to his family. Soon after his diagnosis, Peter claims: “I wasn’t Peter Berry, successful businessman, anymore. […] I was now Peter Berry, living with Alzheimer’s, or Peter Berry, the man that people would feel sorry for or take pity on or avoid in the street because they were too embarrassed to talk to him” (10). Peter’s narrative is aligned with the “personal tragedy” discourse that often accompanies understandings of dementia (Örulv, “Neurodiversity and Dementia,” 247), in a problematic way that conflates Peter with his condition. His frustration is juxtaposed with the problem of social ostracism, contributing to his feelings of diminished value. Most notably, much of Peter’s communication is prevented by a social system doubting the voice of the person with dementia. When at the supermarket with his wife Teresa, Peter observes people’s unwillingness to talk to him: “But with dementia, they assumed I was not able to talk for myself and that somehow, I was no longer Peter. Everything went through Teresa” (67).

Unbeknownst to her, Deb is one of Peter’s enablers. Following what might be called the cultural mainstream approach to Alzheimer’s, Deb’s early narrative contains occasional instances of stigma about the condition, which is centred on ideas of passivity and vulnerability. So, upon meeting Peter, her first impression is that “he didn’t look as if he had dementia. This was my first erroneous assumption about those living with the condition and I shudder even now when I think back to that moment” (42). Deb’s view of Peter is limited both by his external appearance, and by the tiniest pieces of clinical information Deb’s mother shared with her after her grandmother’s Alzheimer’s diagnosis (41). Implicit to Deb’s reasoning here are latent biases reinforcing the idea of a power imbalance between herself and Peter, at least on Deb’s part, which risks affecting the dynamics of their collaboration.

Contrary to this reading, a fanfiction-informed framework highlights the positive impact of Peter and Deb’s partnership on their understanding of dementia and their perceptions of their own and each other’s life stories. The memoir, in fact, hinges on a transformative moment marked by Peter and Deb’s decision to write *Slow Puncture* together. Collaborative life writing, becomes, for them both, a cornerstone of their friendship, to the extent that the resulting memoir is shaped as much by their partnership as their friendship is shaped by their collaboration in this project. Deb recounts moments from a conversation she shared with Peter while they sat together in a café in Framlingham:

“Will you write that down?” he asks. “That was quite good, wasn’t it? Well, I think it was. I don’t really remember it now.”

“It was more than good. Here’s a thing,” I say. “… it was stunning.” (153)

Not only is Peter’s perspective affected by his collaboration with Deb; Deb, too, becomes acutely aware of the influence Peter has on her outlook. During another coffee break, Deb observes: “*Here it comes*, I thought to myself. *Another bite-sized Berry maxim to remember and record*” (103). Insofar as Deb is documenting Peter’s experiences, their partnership might be explained through Couser’s declaredly simplistic model of collaboration in life writing: “There are, of course, different kinds and degrees of collaboration, but in most cases, one member supplies the ‘life’ while the other provides the ‘writing’” (*Vulnerable Subjects*, 36). For Couser, the two partners’ contributions are not just different, but inherently incommensurate.

Yet, the above textual exchanges also hint at the productive interaction that collaborative life writing has on both partners’ lives *irrespective of* their alleged creative roles. Like collaboration in fanfiction – as Thomas observes in her study of adolescent fanfiction writing – working together on *Slow Puncture* offers both partners an enriching experience and a deeper understanding of themselves and each other. Peter and Deb are collaborating to create a common product and, while doing so, both parties gain insight into facets of the other that may otherwise have remained concealed (*Slow Puncture*, 151). On the one hand, in a notable discussion of the collaborative process of producing the memoir, the so-called “story of the story” (Eakin), Deb explains how giving voice to Peter’s dementia created a whole new world for her: “As much as the dementia has stripped Peter bare in so many ways, it seems to feed me, to enrich me and, in a curious twist of events, his dementia has become empowering for me” (x). Peter’s perspective increasingly weaves into Deb’s writing, shaping it because of their close proximity. When considering how to respond to one of Peter’s lapses in memory, Deb reflects: “From Peter’s perspective, surrounding dementia with simple solutions was to alleviate unnecessary pain and angst and so, I had decided, this was what I would do” (125). By assimilating multiple viewpoints, Deb learns to question the apparent fixity of her individual self. Peter’s words also provide her with analogies to include in her own narrative (93), and at times, sentences in the two narratives overlap almost identically. Describing a cycling event they both took part in, Peter writes: “This was what I remembered about the challenge” (129), followed closely by Deb’s own reflection: “This is what I remember about the challenge” (130).

On the other hand, the collaborative writing process and resulting memoir offer Peter a renewed sense of purpose, one that centres on yet transcends his dementia. Initially discouraged by the impossibility of self-fulfilment through personal effort alone, Peter gradually comes to recognise that his realisation lies not in autonomy and independence. While Martina Zimmermann notes that many dementia self-advocates choose narrative forms aligned with their attention span and political intentions as a way to reclaim authority (95-116), Peter embraces interdependence as his own path forward: “When I met Deb and she started to jot down the things I said, I felt so reassured that my words were now being written in stone. It was important for me that my words were not lost but that someone captured them” (77). In a similar way to how fanfiction platforms amplify the voices of both active writers and those who engage more privately in fandom discourse, collaborative life writing offers Peter a platform to articulate the challenges faced by underrepresented minorities and reclaim his voice as a disabled yet active agent. Through collaboration, Peter grows aware of his potential influence not only within his close circle but also on a broader audience who will read his memoir; so, when reflecting on the benefits of cycling, for example, he addresses the reader directly: “So, here is a message, a reality check for everyone out there, not just those of you living with a terminal condition, but for everyone. If you want to do something (and it’s legal and affordable), then I would urge you just do it!” (55).

As the narrative strands become increasingly intertwined, Lindemann’s question *who is the author?* becomes more complex and open to criticism. In particular, we ask – tentatively – whether this question must be framed in the singular. Another way in which *Slow Puncture* challenges individualism and notions of personalised authorship is through its use of section titles. While half of the book’s subchapters are prefaced by titles clarifying the narrator’s perspective – for example, “DEB / (i) / SOUTH LONDON MEETS SAXMUNDHAM” (39) – many others are not, and the use of titles does not strictly correspond to shifts in narrative perspective. Untitled subchapters can still be read from the viewpoint of either Peter or Deb, discernible through contextual clues. When a passage reads, “I had posted on Peter’s Facebook page details of our itinerary” (140), the mention of Peter’s name subtly signals Deb’s viewpoint. At the same time, in the context of our argument, *Slow Puncture* seems to subtly challenge the overemphasis scholars of autobiographical writing have placed on a text’s signature – and its possible iterations within the text – as the irrefutable link to a real person beyond the text, the owner of the story. Instead, much like fanfiction, where pseudonyms often function as the only markers connecting text and contributors, the memoir’s inconsistent clarification of narrative perspectives creates the impression of individual viewpoints becoming increasingly more reductive, while the memoir progressively builds on shared experience. *Slow Puncture* invites readers and critics to focus on the collective creation of the two partners, foregrounding the “true” possibility of collaborative authorship.^2^ In this sense, the memoir belongs to neither Peter nor Deb; it belongs to them both.

### *Somebody I Used to Know:* Against Ventriloquism

Unlike *Slow Puncture, Somebody I Used to Know* is characterised by an apparent absence of multiple speakers. Produced dialogically between Wendy Mitchell and Anna Wharton, the book is, at first glance, a monological account of Wendy’s life with early-onset Alzheimer’s, striving to raise awareness on both the limits and possibilities linked to the condition. The absence of paratextual sections in which all parties come together to give an account of the process that produced this memoir – “the story of the story” as Eakin calls it – strengthens the perception of this work as monological and complies with Lejeune’s belief that in collaborative autobiographies the writer must relay the subject’s experience by discreetly effacing themselves (“The Autobiography of Those Who Do Not Write”, 190). The speaking voice, the memoir clarifies, is that of Wendy (6). However, because of this, *Somebody I Used to Know* risks disregarding Anna’s mediation, which is only briefly mentioned in the book’s Acknowledgements. To use Couser’s words, this memoir is in danger of being “(mis)taken for a transparent lens through which we have direct access to its subject (rather than to its author [*sic*])” (*Vulnerable Subjects*, 38-39).

For Couser, openly acknowledging mediation is important as it illuminates the ventriloquism implicit in collaborative autobiography, which he describes as “the direction in which the voice is ‘thrown’” (*Vulnerable Subjects*, 48). In his view, ventriloquism, when ignored, is akin to forgery on the part of the writer. Nevertheless, this idea, we have suggested, depends on a model that understands the partners’ contributions as disparate and fixed, rather than as part of a dynamic exchange. The very notion of “throwing the voice” implies a one-way movement from subject to writer, which is underscored by the name of certain forms of collaborative life writing, such as as-told-to autobiographies. Conversely, as in *Slow Puncture*, an approach highlighting the productive exchange of collaborative life writing allows us to foreground another way in which the interdependent relation between Wendy and Anna unfolds *within* the text, and helps us reconsider the memoir’s narrative “I” in terms of its possibilities rather than as “a simulation by one person of the voice of another – [which] is always in danger of breaking, exposing conflicts not manifest in solo autobiography” (Couser, *Vulnerable Subjects*, 35).

Particularly interesting, in this respect, are the italicised sections throughout *Somebody I Used to Know*, which serve as personal reflections on Wendy’s life before the onset of dementia: “*You can probably remember the light flashing on the screen, one of the first calls coming in and your turn to answer. / ‘NHS Direct, Wendy speaking, how can I help you?’*” (239). The alternation between regular and italicised passages suggests a shift in narrative flow. Crucially, however, the identity of the narrator in the italicised sections is never clarified, echoing *Slow Puncture*’s deliberate ambiguity in clarifying narrative perspective. If we interpret these italicised sections in line with information from the main narrative, recounted by Wendy, we might assume that she serves as *both* the addressee and the speaker in the italicised sections. A comparison of two passages, one from the main narrative and another from the subsequent italicised section, supports this view. Discussing dementia’s impact on her emotions, Wendy observes, “So I can’t even feel angry, just sad” (250). This comment is followed by an italicised passage on her experience as a mother, concluding with: “*That’s what made you mad then. Now it would just make me sad*” (251).

The subtle shift from second to first person, along with the shared sadness of both narrators, suggests they may indeed be the same person: Wendy. Additionally, mention that the narrator in the italicised sections resides in the same village where Gemma lived (9), resonates with Wendy’s decision to move into “a semi-detached house in the same village as Gemma” (195). From this perspective, the narrator of the italicised sections would be reflecting back on her life before the diagnosis of early-onset Alzheimer’s.

It is here that the veracity of *Somebody I Used to Know* as a first-person account of Wendy’s life may be called into question. The depth of insight promoted by the narrator of the italicised passages contrasts sharply with the reader’s knowledge of Wendy’s cognitive decline as she describes it elsewhere: “What else was out of place? But of course it’s impossible now to remember where it once all stood” (218). In light of this, the level of detail contained in many of the italicised passages, which recount decades-old memories, seems almost ironic: “*You’d make miniature cupcakes* […] *some iced with funny faces in different colours* […] *Some you’d top with marshmallows and edible pink glitter*” (57). In Couser’s words, the memoir’s monological prose “belies the very labor-intensive dialogical process by which it was produced; in fundamental ways, it masks or erases the disability that has so profoundly shaped its subject’s life” (*Vulnerable Subjects*, 39). By not engaging with Anna’s mediation, the monological text is at risk of hypernormalising Wendy’s experience with dementia, to the extent that it omits – or at least seriously under-acknowledges – that its literary transposition was collaboratively shaped. This risk is compounded by the presentation of Wendy as the sole author of *Somebody I Used to Know* on the book cover, overshadowing her role as one of two contributors. Here, a paradox arises: the mediation intended to empower Wendy overshadows her voice, raising issues of power imbalance that complicate questions of authenticity and authorship in this piece of collaborative life writing.

But another interpretation is possible. We want to use the example of fanfiction to explore the discrepancy between the information about Wendy’s dementia presented in the main narrative and the perspective of the narrator in the italicised passages, framing this contrast as productive evidence of Wendy and Anna’s collaboration. As noted by Abby Kirby, the collaboration and active debate among fanfiction contributors is visible in the narrative; this is not condemned, as fanfiction narratives are sites of negotiation of meaning (n.p.). Similarly, *Somebody I Used to Know* is neither less authentic nor less autobiographical because of the challenges posed by Wendy’s dementia, requiring Anna’s contribution; rather, this work is defined precisely by the creative interdependence of the two partners. Couser himself observes that “a model of functional independence requiring unassisted communication would be discriminatory and oppressive; autonomy does not mean or require absolute independence” (*Vulnerable Subjects*, 53). Therefore, the writing process of *Somebody I Used to Know* and the resulting memoir should not be seen as any less significant simply because they are impacted by one of the contributors’ Alzheimer’s. On the contrary, Wendy goes from believing that her fate has been decided for her (93) to recognising the value of interdependence as a tool for self-realisation. As she observes during a conversation with another man living with Alzheimer’s:

“But how will she [the man’s wife] know how to help you if you don’t talk about it?” I said. “And how can you help her to understand if you don’t talk to each other? If you don’t, she’ll just make up her own stories without knowing what’s really going on.” (202)

Interdependence originates in communication and is also present, as Wendy herself acknowledges in an interview to *Granta*, in the dynamics that characterised her working relationship with Anna: “I remember your [Anna’s] expression in your WhatsApp messages”, she explains, “you’d say: ‘You know I’m going to probe today…’” (Mitchell and Wharton n.p.). With regard to this, the frequent questions introducing the italicised passages in *Somebody I Used to Know* can be appraised as a way to admit the collaborative process into the narrative, as textual evidence of Wendy and Anna’s joint endeavour to recall the former’s life both before and after her Alzheimer’s diagnosis. So, the narrator of the italicised passages asks post-diagnosis Wendy, “*Do you remember your first day of work?*” (154), “*Do you remember visiting someone with dementia once, long before your own diagnosis?*” (161), and later, “*Do you remember the teenage years, or is that a period you’d gladly let dementia steal from you?*” (235). From this perspective, Anna’s mediation is essential to Wendy’s ability to speak of her past. But the singular narrative voice is not meant as “a simulation by one person of the voice of another” (Couser, *Vulnerable Subjects*, 35); rather, it becomes the result of entanglements between different subjectivities where the strains of individuality must be (contingently) relinquished. The passages in italics create a space in which Wendy’s post-diagnosis voice is realised communally, and Anna’s writing resists fully taking over the relationship with the reader *as if* she were the subject but can still make her contribution manifest in the midst of an interpersonal relationship of dialogue with Wendy.

In this light, the memoir’s decision not to openly disclose the identity of the narrator in the italicised passages takes on a higher meaning. When juxtaposed with *Slow Puncture*’s omission of the narrator’s identity from half of its subchapter titles, this choice marks a pattern that can be seen as part of both memoirs’ efforts to encourage a potentially productive reading that destabilises conventional approaches to collaborative life writing and resists clear attribution of the story to just one of its contributors, the nominal author. Rather, the ambiguous correspondence between Wendy and the narrator in the italicised sections productively challenges the concept of personalised authorship in this memoir. In the context of literary dementia studies working to reclaim the patient’s voice (Zimmermann), an interdependent approach to *Somebody I Used to Know* does not make Wendy any *less than* she is accustomed to being, as if her identity is somewhat curtailed. Her collaboration with Anna, instead, results in a successful example of “access intimacy,” a term introduced by Mia Mingus “to name the feeling, practice, and politics of interpersonal relationships,” either between disabled people or between disabled and nondisabled people, “as they work to shape the world differently” (Valentine 81).

## Conclusion: Agile Authorship

While the ethics of collaborative life writing warrant careful consideration, the life stories discussed in *Slow Puncture* and *Somebody I Used to Know* highlight the advantages of embracing a non-hierarchical approach to autobiographical collaborations in dementia memoirs, which challenges comfortable notions of personalised authorship and is entrenched in dementia self-advocates’ insistence on interdependence. For people with dementia like Peter and Wendy in particular, decentring individuation creates opportunities for self-expression as well as authorial negotiation. *Slow Puncture* and *Somebody I Used to Know* are only two examples of collaborative dementia memoirs, but they serve as significant illustrations of what Gee calls an “affinity space” (67).

Taking the collaborative model of authorship in action in fanfiction and borrowing from Stuart Murray’s discussion of agility in contexts of health and disability (123-28), we propose the concept of “agile authorship” to encapsulate the approach we have articulated in this article. We contend for the usefulness of the notion of agility to emphasise the exchange-based dynamic underlying most autobiographical collaborations, one that moves fluidly between subjectivities and gives rise to integrally plural – but grammatically singular – narrative voices. In line with fan culture, agile authorship underscores the essential roles of all contributors with and without dementia in the creation of collaborative dementia memoirs. Within this framework, questions like *whose story is this?* and *who is the author?* are no longer framed in terms of individual selves; rather, the focus shifts onto the nuanced entanglements between disabled and nondisabled lives. At the same time, as Murray observes about practising agile scholarship, our approach is inevitably precarious and “can still feel insecure, anxious of its location and even pursued by detractors as it undertakes its work” (125). Such complications, we argue, do not constrain the potential of collaborative dementia memoirs, but underscore the importance of continuing to explore how collaborative dynamics evolve both within the story and among contributors.

## Footnotes

1 In choosing this term, we have found Stuart Murray’s discussion of agility in contexts of health and disability particularly useful (123-28).

2 It is interesting, in this sense, that Couser, while defending the possibility of mutually beneficial collaborative life stories, speaks of “true peers” only with reference to dual autobiographies (*Vulnerable Subjects*, 40), thus suggesting the presence of an imbalance of power in all other forms, however amicable, of life writing.
